# Exploring 3D Human Action Recognition: from Offline to Online

**DOI:** 10.3390/s18020633

**Published:** 2018-02-20

**Authors:** Rui Li, Zhenyu Liu, Jianrong Tan

**Affiliations:** State Key Lab of CAD&CG, Zhejiang University, Hangzhou 310027, China; lirui_2013@yeah.net (R.L.); egi@zju.edu.cn (J.T.)

**Keywords:** action recognition, skeletal sequence, depth map, online segmentation, Kinect

## Abstract

With the introduction of cost-effective depth sensors, a tremendous amount of research has been devoted to studying human action recognition using 3D motion data. However, most existing methods work in an offline fashion, i.e., they operate on a segmented sequence. There are a few methods specifically designed for online action recognition, which continually predicts action labels as a stream sequence proceeds. In view of this fact, we propose a question: can we draw inspirations and borrow techniques or descriptors from existing offline methods, and then apply these to online action recognition? Note that extending offline techniques or descriptors to online applications is not straightforward, since at least two problems—including real-time performance and sequence segmentation—are usually not considered in offline action recognition. In this paper, we give a positive answer to the question. To develop applicable online action recognition methods, we carefully explore feature extraction, sequence segmentation, computational costs, and classifier selection. The effectiveness of the developed methods is validated on the MSR 3D Online Action dataset and the MSR Daily Activity 3D dataset.

## 1. Introduction

Human action recognition has become an active research field due to its promising applications, ranging from robotics to human-computer interaction (HCI) and video surveillance. Although this topic has been extensively investigated over the last two decades and marked progress has been made, it remains one of the most challenging tasks in computer vision. Human action recognition can be classified into different categories according to different criteria [1,2,3]. We have no intention of providing a comprehensive survey in this paper. Instead, we concentrate on 3D single-view- and single-person-based human action recognition.

In the past, expensive wearable 3D motion-capture devices, such as the Vicon optical system, were inconvenient to deploy and only installed in a laboratory. Nowadays, 3D markerless motion capture devices entered millions of ordinary families with the advent of consumer depth sensors, e.g., Kinect, Xtion, and Leap Motion. Compared to 2D images, depth maps obtained by these sensors reflect pure geometry shapes of objects, and are insensitive to color, texture, and illumination changes. The progress in human pose estimation from depth maps [4] markedly encourages 3D skeletal sequence-based action recognition study.

Recent 3D human action recognition methods use skeletal sequences [5,6,7,8,9,10,11,12,13,14], depth maps [15,16,17,18,19,20,21], both of skeletal sequences and depth maps [22,23,24], or RGB-D sequences [25,26] as motion data. They can be divided into offline [5,6,7,8,9,10,15,16,17,18,19,20,22,23] and online [11,12,13,14,21,24,25,26] action recognition methods. The former requires a segmented sequence for feature extraction and classification, whereas the latter continually predicts action labels as an unsegmented stream sequence proceeds. Particular challenges of online action recognition primarily stem from two aspects: (i) low observational latency, i.e., sequentially recognizing actions with a delay of a few frames, and (ii) low computational latency, i.e., the system computational time should not go beyond the real-time requirement.

From an application perspective, online action recognition is obviously more accordant to practical scenes, especially for real-time HCI where a quick response is needed, e.g., interactive entertainment. High latency causes the system’s feedback to lag behind user actions and, thus, degrades the interactivity of user experience. Most existing methods are originally proposed for offline action recognition. In contrast, only a small number of methods are specifically designed for recognizing stream sequences, although a minority of offline methods can be extended to work in an online fashion.

The recognition data type of online methods is frame-wise or continuous frames within a short duration, i.e., a sub-sequence. Accordingly, online methods can be categorized into frame-wise-based ones like [11,24] and sub-sequence-based ones like [12,13]. Due to the difference of the recognition data type, how to extend an offline method to online applications is unclear. Some offline methods are rooted in complicated energy functions that need a time-consuming optimization process, which also leads to the extended infeasibility. Despite this, existing research on offline action recognition still provides plenty of valuable guidance for developing an online method.

A closely related topic to online action recognition is human motion segmentation that aims to segment a continuous sequence into disjointed sub-sequences so that each of these is corresponding to an action primitive [27,28]. Kernelized temporal cut (KTC) [29], a sequential motion segmentation algorithm, has been successfully applied to online action recognition [13]. However, motion segmentation algorithms like [27,28] are limited to offline applications, because they operate on a segmented sequence with high computational costs. Hence, online action recognition is not a problem that simply combines human motion segmentation and offline action recognition. To reduce the discussion scope, we do not consider borrowing techniques or descriptors from studies on human motion segmentation.

The contributions of our work are that we develop two different types of online action recognition methods. For the first type, the frame-wise feature is constructed by pairwise relative joint positions and Local Occupancy Patterns (LOPs) [22]. The K-SVD [30] is utilized to extract a class-specific dictionary for each action. In conjunction with the learned dictionaries, three Regularized Linear Regression (RLR) techniques are used to achieve frame-wise action recognition, including the Lasso [31], Ridge [32], and Elastic Net [33]. To prevent the sudden change of the predicted action labels, we propose a temporal smoothness scheme. The first method differs from existing offline methods in terms of the feature construction, dictionary learning algorithm, frame-wise recognition, and temporal smoothness. The specific differences will be highlighted by comparing with existing methods in Section 2.

The second of our methods is built on the depth motion map [16,17,18] that was originally proposed for offline action recognition. The depth motion map is extracted from a segmented depth sequence, which hinders its application to online action recognition. For this problem, we propose an offline random segmentation algorithm used at the training stage, and an online sequential segmentation algorithm used at the test stage. The depth motion map only characterizes the dynamic property and has a very limited discriminative power for static actions. In view of this, we introduce the skeletal position to characterize static actions. The depth motion map completely loses temporal information, so it cannot distinguish actions whose differences lie in the order of human poses. For this problem, the skeletal velocity is incorporated to feature vectors. The skeletal position and velocity jointly serve as complementary features.

Our two methods are very representative in that: (i) the first type builds action recognition models with skeletal sequences as the main descriptor and depth maps as the auxiliary one, whereas the second type is just on the contrary. (ii) The first type achieves frame-wise action recognition, whereas the second type achieves sub-sequence-level action recognition. This is the first time that the two types of methods are explored in the same work.

The rest of the contents are organized as follows. Section 2 introduces related studies on action recognition. In Section 3, the two developed methods are elaborated. Experiments on the MSR 3D Online Action dataset and MSR Daily Activity 3D dataset are performed in Section 4. Finally, conclusions are presented in Section 5.

## 2. Related Studies

### 2.1. Offline Action Recognition

From a perspective of feature extraction, existing offline action recognition methods can be classified into handcrafted feature-based methods like [16] and deep learning-based methods like [19]. Currently, deep learning-based methods have achieved competitive, or even superior, performance compared to handcrafted feature-based methods, but they have two significant disadvantages inherited from deep learning. The first one is that they particularly require the support of expensive GPUs. The second one is that they are data-hungry, i.e., they rely on a tremendous amount of training data. For action recognition, acquisition and annotation of depth maps are not very easy tasks. Benefitting from the end-to-end training mechanism, the advantage is that researchers do not need to struggle with specific feature design and selection. Instead, researchers mainly concentrate on the design and optimization of a deep network.

For handcrafted feature-based methods, they are just contrary to deep learning-based methods. They generally do not rely on expensive GPUs, and also do not need too much training data. However, researchers need make an effort to carefully explore a variety of features. Since our methods are based on handcrafted features, we only mention a few handcrafted feature-based methods in the following.

Ellis et al. [7] introduced a latency-aware learning formulation to train a logistic regression-based classifier. The classifier determines canonical poses and uses these to robustly recognize actions with a short delay. Since Ellis et al. do not provide a method to detect action transition, we think that their method is still an offline one.

Yang et al. [10] proposed a type of features by adopting the differences of human joints in both temporal and spatial domains. Principal component analysis (PCA) was applied to the differences to generate EigenJoints. Yang et al. found that 15–20 frames were sufficient for the EigenJoints to obtain comparable results to that using an entire sequence (about 50 frames).

Xia et al. [20] extended local spatiotemporal interest points (STIPs) from RGB videos to depth maps, called DSTIPs. They built a novel depth cuboid similarity feature (DCSF) to describe the local 3D depth cuboid around the DSTIPs with an adaptable supporting size. The DSTIP + DCSF can also be applied to motion segmentation and human detection.

The methods proposed in [10,20] have been implemented for online action recognition by Yu et al. [24], but the achieved accuracies are relatively low. In our experiments, the two methods serve as the baselines. The aim is to demonstrate that directly extending an offline method to stream sequences cannot ensure attractive performance.

The depth motion map has been first proposed by Yang et al. [16]. The authors utilized the histograms of oriented gradient (HOG) to encode the depth motion map. Chen et al. [17] employed an *l*_2_-regularized collaborative representation classifier to directly classify the depth motion map, whereas, in [18], they encoded the depth motion map using the local binary pattern (LBP) histograms.

As far as we know, the depth motion map has never been applied to online action recognition. The largest drawback is that it requires a segmented sequence, but for online action recognition, frames of a stream sequence are sequentially observed. Moreover, it loses the temporal information and has very limited discriminative power for static actions. Chen et al. [17] artificially removed the first five frames and the last five frames from each sequence because, in their evaluation dataset, all the actions of interest were dynamic, and humans were mostly standing at the beginning and the end. This operation discards the poses that do not contribute to the motion characteristic, but is obviously infeasible for online action recognition. We solve these problems, and successfully extend it to online action recognition. Our solutions may also inspire the improvement of offline depth motion map-based methods, because the latter drawbacks exist in both offline action recognition and online action recognition.

Traditional depth motion map-based methods like [16,17,18] do not specially extract the human foreground, because the background of the scene is clean in the evaluation dataset, and the human foreground extraction can be easily accomplished by depth-distance thresholding. The depth motion map-based method proposed in [19] converts a depth map into a pseudo-color image, so the entire scene is encoded. Considering that an estimated skeleton is strictly synchronized with a depth map [4], we propose a human foreground extraction approach by the rectangular envelope of the skeleton. We believe that our depth motion map-based methods can benefit from this segmentation.

Wang et al. [22] characterized actions with a novel actionlet ensemble model that represented the action conjunctive structure by capturing the correlations of the joints. They proposed the LOP to describe the depth appearance in the neighborhood of the joints. We borrow the LOP to characterize the object shape, but besides this, our methods are totally different from [22].

Yao et al. [34] employed the Elastic Net as a model classifier. Their method is oriented to a single static RGB image. In their method, the Elastic Net is used to learn a set of sparse bases of action attributes and parts. In contrast, we attempt the more general RLR in our first method, and the Elastic Net is merely a special case of the RLR. In terms of the action data type, application scene, and feature descriptor, our methods are also very different from [34]. In addition, the confidence derived from the reconstruction error and the temporal smoothness scheme in our first method are specifically designed by us for online action recognition.

### 2.2. Online Action Recognition

It has been observed that human actions can be well modeled through a few key poses [11] or a set of key joints [15]. When performing certain actions, a person often has to go through some poses that are shared by other actions. This implies that the training data are weakly supervised: not all the frames in a training sequence specifically belong to that action. Irrelevant and neutral configurations, e.g., standing poses, are of not much help for modeling action discriminations, or even have a negative impact. A natural solution is to develop a compact, but effective, representation.

Mining discriminative poses or motion primitives is such a simple way. Zhu et al. [12] divided a continuous sequence into pose feature segments and motion feature segments with an online segmentation method. They computed probabilities that each feature segment can be labeled as key poses or atomic motions in a matching process. An online variable-length maximal entropy Markov model was proposed to make classification. Since our feature vector simultaneously characterizes the dynamic property and the static property, unlike [12], we do not divide action primitives as key poses and atomic motions.

Zanfir et al. [11] proposed a non-parametric descriptor that incorporated both pose information and differential quantities of the joints. A modified *k-*NN classifier was employed to discover the most discriminative poses. The descriptor enables low-latency action recognition on unsegmented sequences. The classification decision function of [11] is the value of accumulated votes for the winning class divided by the total number of votes for all the actions. Different from this decision function, our classification decision scheme with temporal smoothness is the accumulated confidence within sliding windows, which considers the coherence of the action being performed and the transition of adjacent actions.

Eum et al. [21] described features using depth motion energy images and motion history images. A spotter model was proposed to filter meaningless actions from continuous actions and to identify the precise start and end points of actions.

Yu et al. [24] presented a middle level feature representation to define primitive ordinal patterns, referred to as orderlet. The orderlet can be applied to human skeletons to encode inter-joint coordination, as well as depth maps to encode action-related object shape. A mining algorithm was proposed to discover the most representative orderlets, and AdaBoosting was used to combine these. The orderlet relies on manually labeled data to describe potential object positions. In contrast, all the features of our methods are automatically extracted. We should note that low-latency, high-accuracy, and automatically-completed requirements are equally important for online action recognition.

Wu et al. [26] attempted to model human activities comprising multiple actions in an unsupervised setting. Their model learns high-level action co-occurrence and temporal relations, which can be applied to unsupervised online action segmentation and recognition, as well as forgotten action detection. Their method is a rare exception that performs online action recognition in an unsupervised manner. Our methods are developed for supervised action recognition.

Evangelidis et al. [14] addressed continuous gesture recognition in a labeling framework. Each frame must be assigned a label. A global energy function was formulated to ensure a piece-wise constant labeling. They proposed a dynamic programming solver that sought the optimal path in a recursive manner. Fisher vectors were introduced to encode skeletal descriptors.

Fanello et al. [25] mapped each depth region of interest into a feature space with a combination of 3D histogram of flow (3DHOF) and global histograms of oriented gradient (GHOG) models. The 3DHOF+GHOG descriptor was processed via sparse coding. They proposed an online segmentation algorithm that worked concurrently with a SVM. So-called real-time action recognition in [25] is essentially online gesture recognition that only considers hand movements. Both [25] and [14] focus on gesture recognition, whereas we pay attention to the actions that are described through full-body poses and movements.

Due to the weak supervision of training data, current methods follow a bag-of-words paradigm to obtain a condensed representation [11,12,21,24,26]. The first method also continues in this vein. Different from [11] using the *k*-NN classifier, [12] using the GMM, [21,26] using the *k*-means, and [24] using the orderlet mining algorithm, we use the easy-to-use K-SVD to accomplish the task of dictionary learning. The raw feature, application scene, and subsequent modeling of these methods are also very different from those of our methods. We refer interested readers to the past work mentioned above, and focus on the innovations particular to our methods.

Naturally, not all the methods follow the bag-of-words paradigm. Gong et al. [13] decomposed online action recognition into two sub-problems under a structured time series framework, including sequence segmentation and sequence alignment. They employed the KTC [29] to detect action transition in real time. The KTC was capable of capturing action units, e.g., periodic motions. Dynamic manifold warping (DMW) [35], a spatiotemporal alignment algorithm, was used to measure the similarity of segmented sub-sequences. This pure structured time series-based method has difficulties in recognizing actions involved with objects.

Devanne et al. [36] proposed a framework for analyzing and understanding human behavior. They first decomposed the full motion into short temporal segments by shape analysis of human poses. Each segment was then characterized by human motion and depth appearance around hand joints. The sequence of temporal segments was finally modeled through a dynamic naïve Bayes classifier (DNBC) that captured the dynamics of elementary motions. This method can be applied to both gesture recognition and activity recognition.

## 3. Proposed Methods

The content of Section 3 is organized as follows: Skeletal sequence preprocessing is introduced in Section 3.1. The K-SVD- and RLR-based methods are presented in Section 3.2. The depth motion map-based methods are presented in Section 3.3. The skeletal sequence preprocessing of the K-SVD and RLR-based methods is the same as that of the depth motion map-based methods.

### 3.1. Skeletal Sequence Preprocessing

**Figure 1:**
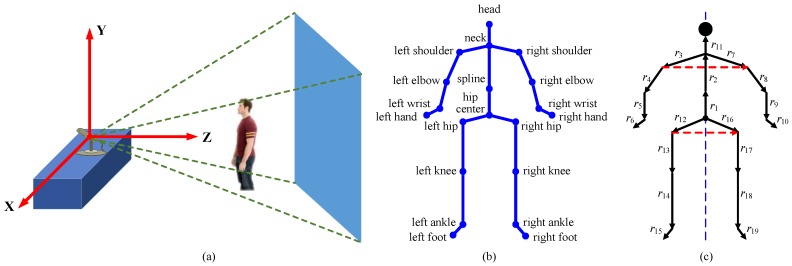
Skeleton and preprocessing. (**a**) World coordinate system of the Kinect. (**b**) Twenty joints provided by the evaluation datasets. (**c**) Nineteen limb lengths represented by *r_i_* (*i* = 1, 2, ..., 19). The two vectors indicated by the red dashed arrows are constructed for viewpoint calibration. The blue dashed line indicates the symmetry of the human body.

The Kinect is used as a motion capture device in our evaluation datasets. Figure 1a shows its world coordinate system. The datasets provide human pose estimation results with 20 joint positions, as shown in Figure 1b. The skeletal sequence is simultaneously estimated from a depth map. Following our previous work on human motion segmentation [28], we perform the following skeletal sequence normalizations:

#### 3.1.1. Denoising

We smooth each coordinate along the time dimension with a 5 × 1 Gaussian filter (*σ* = 1). Gaussian smoothing produces a lag of a few frames but does not impact the overall performance.

#### 3.1.2. Translation Invariance

All the joint coordinates are transformed from the world coordinate system to a person-centric coordinate system by placing the hip centered at the origin.

#### 3.1.3. Human Body Size Invariance

Human subjects have variations in body size that are not relevant to the action class. We impose the same limb lengths for poses to compensate for anthropometric differences. First, we obtain the limb lengths by taking a skeleton in training data as a reference. Each limb is defined by any two linked joints as shown in Figure 1c. Assuming that **r** = [*r*_1_, *r*_2_, …, *r*_19_] is the expected limb lengths, we adjust **r** to have a unit *l*_2_-norm. Considering the symmetry of the human body (as shown in Figure 1c), if the *i*th limb and *j*th limb are symmetrical, we set *r_i_* and *r_j_* to be their average. Second, for each frame, we start from a hip joint, move forward the branches of the kinematic tree associated with the joints (search order as shown in Figure 1c), and successively modify the joint locations accordingly, such that the *i*th limb length becomes equal to *r_i_*. Since the direction vectors are preserved, the joint angles or human poses are not modified.

#### 3.1.4. Viewpoint Invariance

As shown in Figure 1c, two vectors are constructed by left-right shoulder joints and left–right hip joints. We leverage their ground plane projections, i.e., **n***_s_* = [*s_xr_* – *s_xl_*, *s_zr_* – *s_zl_*] and **n***_h_* = [*h_xr_* – *h_xl_*, *h_zr_* – *h_zl_*], where *s_xr_* and *s_xl_* denote the **X** coordinates of the right and left shoulder joints, respectively. The meanings of the other symbols are obvious. The view calibration vector is formulated by **n** = (n*_s_* + n*_h_*) = [*n_x_*, *n_z_*]. The calibration angle is obtained according to θ = *arctan*(*n_z_* / *n_x_*). The **Y**-axis rotation matrix obtained by substituting θ renders **n** parallel to the **X**-axis of the Kinect.

### 3.2. K-SVD- and RLR-Based Methods

**Figure 2:**
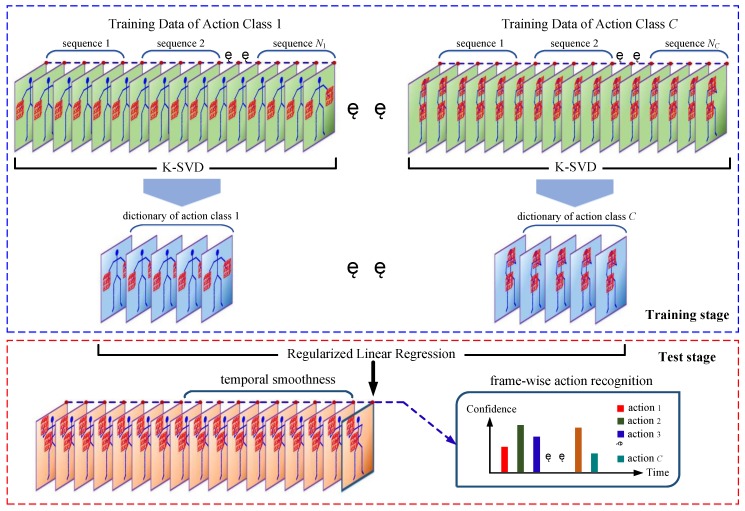
General pipeline of the K-SVD and RLR-based methods. The red wireframes around human hands indicate the LOP feature. *N_i_* is the number of training sequences of the *i*th action. The upper part describes the process of dictionary learning by the K-SVD at the training stage. The lower part describes frame-wise action recognition achieved by the RLR at the test stage. In conjunction with the *C* learned dictionaries, each test frame is predicted by *C* confidences associated with the *C* actions.

The pipeline of the K-SVD and RLR-based methods is shown in Figure 2. For this type of method, the human pose is described by pairwise relative joint positions, which is introduced in Section 3.2.1. The object shape is described by the LOP feature, which is introduced in Section 3.2.2. The frame-wise feature vector is the concatenation of the human pose vector and object shape vector. In this way, each frame of depth sequences is described by a frame-wise feature vector. For each action, the K-SVD is utilized to learn a class-specific dictionary from training sequences, which is introduced in Section 3.2.3. In conjunction with the learned dictionaries, frame-wise action recognition is achieved by the RLR, which is introduced in Section 3.2.4. Since frame-wise action recognition is not robust, a temporal smoothness scheme is proposed in Section 3.2.5.

#### 3.2.1. Pairwise Relative Joint Positions

Finding a suitable feature representation is an important step for designing an action recognition system. Vemulapalli et al. [6] compared four alternative skeletal representations, including joint positions, pairwise relative joint positions, joint angles, and individual body part locations. Compared to the joint positions, the pairwise relative joint positions capture more complex motion relations. For the joint angles, we need to introduce Euler angles or quaternions. If we use the individual body part locations, actions are modeled as curves in the Lie group. To make a classification, we have to perform interpolation on the Lie group, and map all the curves from the Lie group to the Lie algebra. This time-consuming process impedes the real-time implementation.

With a comprehensive consideration of the description capability and the real-time performance, the pairwise relative joint positions are adopted to describe a human pose at temporal index *t*, i.e.:

(1) $$r_{t}={\left[||r_{t,\;1}-r_{t,\;2}|{|}_{2}^{2},\ldots ,||r_{t,\;i}-r_{t,\;j}|{|}_{2}^{2},\ldots ,||r_{t,\;19}-r_{t,\;20}|{|}_{2}^{2}\right]}^{T}\;,$$

where r*_t,i_* = (*r_t,ix_*, *r_t,iy_*, *r_t,iz_*) is the *i*th normalized joint position at temporal index *t* (1 ≤i, j≤20 and i≠j), and ${\left[\cdot \right]}^{T}$ denotes the matrix transformation. We adjust **r***_t_* to have a unit *l*_2_-norm length by:

(2) $$p_{t}=\frac{r_{t}}{||r_{t}|{|}_{2}}.$$

The purpose of Equation (2) is to balance the weight influence of the subsequent concatenation with the object shape feature vector.

#### 3.2.2. Local Occupancy Patterns

For human-object interaction (HOI) actions, introducing object shape information is helpful to enhance action distinctions. We utilize the LOP [22] to characterize the 3D point cloud around the joints. Wang et al. [22] applied the LOP to all 20 joints. The resultant high dimension apparently increased the computational cost. Considering that the HOI mainly occur between hands and objects, we apply the LOP to the left-right hand joints only, as the red wireframes shown in Figure 2. Specifically, the local region of each hand joint is partitioned into $N_{x}{\times N}_{y}{\times N}_{z}$ spatial grids. Each bin *b_xyz_* of the grid is of size (*S_x_*, *S_y_*, *S_z_*) pixels. The number of the points that fall into *b_xyz_* is counted. [22] uses a sigmoid function to normalize *b_xyz_*. To increase the monotonicity, we adopt an exponential function instead. In this way, the local occupancy information of *b_xyz_* is defined as:

(3) $$o_{xyz}=\exp(\mu \sum_{q\in bin_{xyz}}I_{q}),$$

where I*_q_* = 1 if the point cloud has a point in the location *q* and I*_q_* = 0 otherwise, and μ is a constant coefficient that is experimentally set to be 0.05. The LOP feature vector consists of the feature *o_xyz_* of all the bins. Let **o***_t,l_* and **o***_t,r_* denote the LOP feature vectors of the left and right hand joints at temporal index t, respectively. The object shape is described by the concatenation of **o***_t,l_* and **o***_t,r_* with a unit *l*_2_-norm length, i.e.:

(4) $$o_{t}=\frac{{\left[o_{t,\;l}^{T}，\;o_{t,\;r}^{T}\right]}^{T}}{||{\left[o_{t,\;l}^{T}，\;o_{t,\;r}^{T}\right]}^{T}|{|}_{2}}.$$

The final frame-wise vector **f***_t_* is the concatenation of the human pose vector **p***_t_* and object shape vector **o***_t_*:

(5) $$f_{t}={\left[p_{t}^{T},o_{t}^{T}\right]}^{T}.$$

In our experiments, the size of the spatial grids $N_{x}\times N_{y}\times N_{z}$ is set as 6 × 6 × 6 without optimization. If the actions to be recognized are not involved with the objects, the object shape vector should be removed from the final frame-wise vector, i.e., **f***_t_* = **p***_t_*.

#### 3.2.3. Dictionary Learning

We use the K-SVD to learn a class-specific dictionary for each action. Each dictionary is composed of condensed features with a good representative power, thereby reducing the redundant pose configurations. For the *i*th action, we decompose its *N_i_* training sequences into a large number of frames, and then arrange them as a sample matrix **Y***_i_*:

(6) $$Y_{i}=[y_{1,\;1},y_{1,\;2},\;\ldots ,\;y_{N_{i},\;1},y_{N_{i},\;2},\ldots ],$$

where **y***_j, k_* is the *k*th frame-wise feature vector of the *j*th sequence, and 1 < *j* < *N_i_*:

(7) $$y_{j,\;k}=\frac{f_{j,\;k}}{{\left||f_{j,\;k}|\right|}_{2}},$$

where **f***_j, k_* is defined by Equation (5). The unit normalization of **f***_j, k_* is due to the requirement of solving the K-SVD [30]. The atoms of the learned dictionaries serve as the predictors of the RLR.

In our experiments, the K-SVD is implemented using the source code of the public SMALLBox [37]. The atom number K is optimized by two-fold cross validation on the training set. Without specific notes, the other parameters of our methods are also optimized in this manner.

#### 3.2.4. Frame-Wise Action Recognition

At the test stage, we consider how to use the learned dictionaries to approximate a test frame, and how to recognize using the representation coefficients. We attempt to use the RLR to achieve frame-wise action recognition. A typical RLR technique with combined *l*_1_ and *l*_2_ norm penalty terms is defined as:

(8) $$\hat{\beta }=\underset{\beta }{\arg\min}\{||z-V\beta |{|}_{2}^{2}+\gamma_{1}\sum_{j=1}^{p}\beta_{j}+\gamma_{2}\sum_{j=1}^{p}\beta_{j}^{2}\},$$

where **z** is the response, **V** is composed of *p* predictors, $\hat{\beta }={\left({\hat{\beta }}_{1},{\hat{\beta }}_{2},\ldots ,{\hat{\beta }}_{p}\right)}^{T}$ is the representation coefficients produced by the fitting model, $\gamma_{1}$ is the *l*_1_-norm regularization parameter, and $\gamma_{2}$ is the *l*_2_-norm regularization parameter. When $\gamma_{2}$ equals to 0, but $\gamma_{1}$ does not, Equation (8) is the just the famous Lasso [31]. When $\gamma_{1}$ equals 0 but $\gamma_{2}$ does not, Equation (8) is called the Tikhonov regularization or Ridge [32]. When both $\gamma_{1}$ and $\gamma_{2}$ do not equal 0, Equation (8) is referred to as the Elastic Net [33]. When both $\gamma_{1}$ and $\gamma_{2}$ are equal to 0, Equation (8) degrades into the ordinary least squares (OLS) estimate. It is well known that the OLS does poorly in both prediction accuracy and model interpretation, so we do not consider this case.

For the Ridge, it has an analytic solution. Normally, the Tikhonov regularization matrix in the analytic solution is an identity matrix. Following [17], we empower a sample distance weight to its diagonal elements. Least angle regression (LARS) [38] is an effective algorithm to solve the Lasso, which is adopted by our methods. We use LARS-EN [33], which is a modified version of LARS, to obtain the solution of the Elastic Net. We use the source code of the public SMALLBox [37] to obtain the solutions of the Lasso and Elastic Net.

We first take the Ridge as an example to illustrate our frame-wise action recognition. Assume that **D***_i_* is the dictionary of the *i*th action learned from **Y***_i_* of Equation (6), and *C* is the number of the actions. **D** is the concatenation of the *C* dictionaries:

(9) $$D=[D_{1},\;D_{2},\cdots ,\;D_{C}].$$

In this case, **D** is equivalent to **V** of Equation (8), and accordingly **z** of Equation (8) represents a test frame. We still assume that $\hat{\beta }$ is the solution of Equation (8):

(10) $$\hat{\beta }={\left[{\hat{\beta }}_{1}^{T},\;{\hat{\beta }}_{2}^{T},\cdots ,{\hat{\beta }}_{C}^{T}\right]}^{T},$$

where ${\hat{\beta }}_{i}$ denotes the corresponding coefficients of **D***_i_*. The reconstruction error *e_i_* using **D***_i_* to approximate **z** is defined in a squared *l*_2_-norm sense:

(11) $$e_{i}=||z-D_{i}{\hat{\beta }}_{i}|{|}_{2}^{2}.$$

In essence, **z** is approximated using a linear combination of the atoms from **D***_i_*, and ${\hat{\beta }}_{i}$ reflects the representation weight of these atoms. The smaller the value of *e_i_* is, the more likely **z** belongs to the *i*th action. We derive a confidence *conf_i_* for the *i*th action by the reciprocal of *e_i_*, and then make the confidence range from 0 to 1 by normalizing the total confidences of all the actions:

(12) $${conf}_{i}=\frac{1/(e_{i}+\varepsilon )}{\sum_{j=1}^{C}1/(e_{j}+\varepsilon )},$$

where ε is a small positive number to avoid divisions by zero, which is set to be 10^−6^. In this way, **z** is predicted by the *C* confidences associated with the *C* actions. *conf_i_* quantitatively estimates the possibility that **z** belongs to the *i*th action. If there exist at least one action index *j* (i≠j) that make ${conf}_{i}\approx {conf}_{j}$, we think that **z** is ambiguous for recognition. In this case, we can set a threshold to reject recognition. Conversely, if *conf_i_* is much larger than any *conf_j_*, we think that the action membership of **z** is quite clear. Naturally, the action class of **z** is determined by the maximum confidence, i.e.:

(13) $$class\{z\}=\underset{i}{arg\;max}\{conf_{i}\}.$$

As far as the Lasso and Elastic Net, the case is different from the Ridge, because their solutions are sparse [31,33,39], i.e., many entries in the solution vector equal to zero. If we totally follow the computational procedures of the Ridge, for most actions, the reconstruction error *e_i_* computed by Equation (11) will be a meaningless result, namely *e_i_* = ${\left||z|\right|}_{2}^{2}$. Hence, we cannot expect to obtain all the coefficients {${\hat{\beta }}_{i}$} at once. Instead, the reconstruction errors associated with the *C* actions need to be separately computed: at the *i*th time, **z** is approximated using **D***_i_*. Specifically, **D***_i_* substitutes **V** of Equation (8). Accordingly, ${\hat{\beta }}_{i}$ is obtained by solving Equation (8), i.e., ${\hat{\beta }}_{i}$ is equivalent to $\hat{\beta }$ of Equation (8) at this time. When all the coefficients {${\hat{\beta }}_{i}$} are obtained, the reconstruction error and confidence are computed by Equations (11) and (12), respectively. It is the sparsity of the solution that makes the computational procedures of the Lasso and Elastic Net different from those of the Ridge. The Lasso and Elastic Net lead to a sparse representation, whereas the Ridge leads to a collaborative representation. Note that the aforementioned computational procedures of the Lasso and Elastic Net are developed by us, and we do not refer to any other work.

For convenience, the three methods derived from the Lasso, Ridge, and Elastic Net are referred as K-SVD & Lasso, K-SVD & Ridge, and K-SVD & Elastic Net, respectively.

#### 3.2.5. Temporal Smoothness

The classification decision Equation (13) completely isolates the current frame, without considering the confidences of its neighbor frames. Despite Equation (13) enabling the action label to be output without a delay, the action label may change even in a very short duration. We propose the following temporal smoothness scheme to prevent this sudden change:

**Step 1**. Assume that *i* = 1, 2, …, *C*-1 is the *i*th label of actions of interest, and *C* is the label of meaningless or undefined actions. *confThresh* is a preset threshold. *seq*-1 and *seq*-2 denote two sub-sequences within two adjacent sliding windows, and *seq*-2 temporally follows behind *seq*-1. The two windows have the same size, which equals to the sliding step. The reference value of *confThresh* is 2/*C*.
**Step 2**. ${conf}_{i}^{k}$ ← compute the average confidence of the *i*th action of *seq*-*k* (*k* = 1, 2 and *i* = 1, 2, …, *C*).
**Step 3**. *class*{*seq*-*k*} ← determine the action label of *seq*-*k* according to $\underset{i}{arg\;max}\{{conf}_{i}^{k}\}$ (*k* = 1, 2).
**Step 4**. If *max*{${conf}_{i}^{k}$} < *confThresh*, *class*{*seq*-*k*} ← *C* (*k* = 1, 2).
**Step 5**. If *class*{*seq*-1} = *class*{*seq*-2} **| |** *class*{*seq*-1} = *C* **| |** *class*{*seq*-2} = *C*, return *class*{*seq*-*k*} (*k* = 1, 2). Otherwise, *class*{*seq*-1} ← *C*, and then return *class*{*seq*-*k*} (*k* = 1, 2).

Our classification decision scheme is based on two considerations: (*i*) the undefined action label will be assigned to a sub-sequence if the evidence of the actions of interest is not clear enough, as done in Step 4. (*ii*) We hypothesize that there exists no sudden changes between consecutive actions, so two sliding windows are introduced to consider the coherence of the action being performed and the transition of the adjacent actions, as done in Step 5.

### 3.3. Depth Motion Map-Based Methods

**Figure 3:**
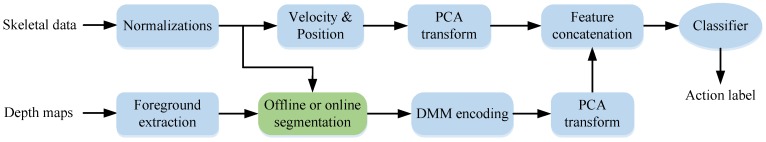
Training and test processes of the developed depth motion map-based methods. The difference between the training and the test processes lie in the sequence segmentation step that is marked in green.

The pipeline of the depth motion map-based methods is shown in Figure 3. Except the sequence segmentation step that is marked with green color in Figure 3, the training process and test process are the same. The depth motion map is introduced in Section 3.3.1. Human foreground extraction from a depth map is introduced in Section 3.3.2. An offline segmentation algorithm is proposed to generate a collection of sub-sequences from training sequences. At the test stage, a sub-sequence must be sequentially segmented from a stream sequence, so we propose an online segmentation algorithm. As described in Section 3.3.1, each sub-sequence is encoded to be a depth motion map. The two segmentation algorithms are described in Section 3.3.3. Due to the inherent drawbacks of the depth motion map, the skeletal velocity and position are introduced as complementary features. The feature fusion process is described in Section 3.3.4. After the feature fusion, two classifiers are attempted to perform sub-sequence-based action recognition in Section 3.3.5.

#### 3.3.1. Depth Motion Map

**Figure 4:**
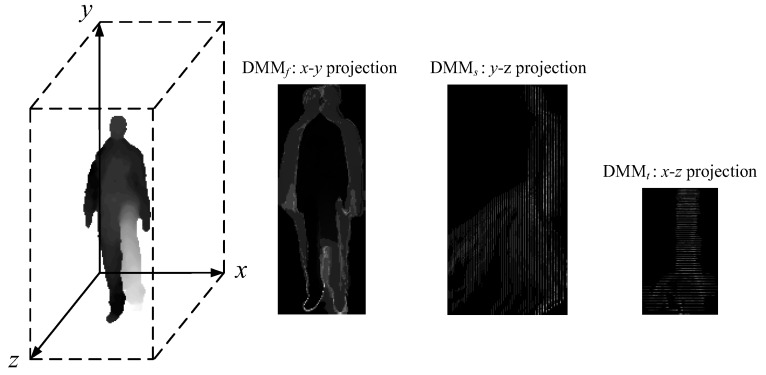
DMM*_V_* generated from a “forward kick” sequence.

The depth motion map was first proposed by Yang et al. [16], and then it is adopted by follow-up methods [17,18,19]. All these methods are developed for offline action recognition. The depth motion map is generated by projecting depth maps onto three orthogonal Cartesian planes and accumulating motion energy across an entire sequence. More specifically, for a depth sequence with *N* frames, DMM*_v_* is obtained by:

(14) $${\mathrm{DMM}}_{v}=\sum_{i=2}^{N}|{\mathrm{map}}_{v}^{i}-{\mathrm{map}}_{v}^{i-1}|,$$

where *v* ∈ {*f*, *s*, *t*} denotes front, side, or top projection views, and ${\mathrm{map}}_{v}^{i}$ denotes the depth frame at temporal index *i*. The depth motion map definition of Equation (14) is given by Chen et al. [17], which is slightly different from that given by Yang et al. [16]. Figure 4 provides an example of the DMM*_v_* generated from a “forward kick” sequence.

The generation process of the depth motion map explains why we claim that applying the depth motion map to online action recognition is unclear: (i) the depth motion map requires a segmented sequence for projection, but for online action recognition, frames are gradually observed as a stream sequence proceeds. How to train using segmented sequences and how to truncate a test sub-sequence to perform action recognition are still unsolved problems. (ii) The depth motion map characterizes the dynamic property of human actions through the accumulated absolute difference. It has a very limited discriminative power for static actions, e.g., “reading books” and “sitting”. (iii) The depth motion map loses the temporal information, so it is incapable of distinguishing actions whose differences only lie in the order of human poses, e.g., “stand up” and “sit down”. Our solutions to the three drawbacks will be given in Section 3.3.3 and Section 3.3.4.

#### 3.3.2. Human Foreground Extraction

**Figure 5:**
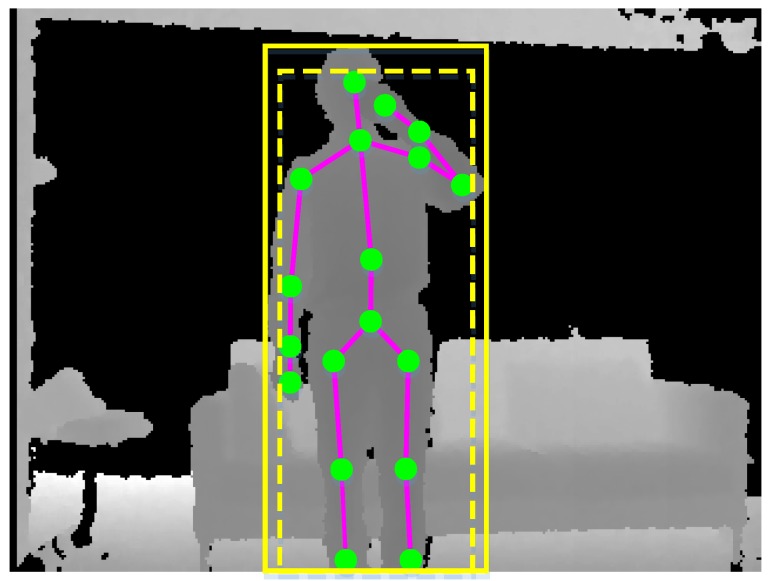
Human foreground extraction from a depth map using the rectangular envelope of the skeleton.

Humans only occupy a small part of the whole scene. It is necessary to extract humans from the noisy background environment, thereby reducing the negative impact of irrelevant objects. We note the fact that skeletal data are simultaneously estimated from depth maps [4], so we utilize skeletal data to segment the region of interest. For a depth map, we first obtain the rectangular envelope of the skeleton in the **X**–**Y** plane, as the yellow dashed rectangle shown in Figure 5. We then slightly enlarge the rectangular envelope along the width and height with a fixed ratio, as the yellow solid rectangle shown in Figure 5. The final rectangular envelope will be cropped if it exceeds the border of the depth map. The points that are far from the skeleton are filtered out through depth distance thresholding.

#### 3.3.3. Offline and Online Segmentation

As far as we know, the skeletal velocity has never been combined with the depth motion map before our work. Assume that **s***_t_* is the normalized skeletal positions at temporal index *t*:

(15) $$s_{t}={\left[p_{2,x}^{t},p_{2,y}^{t},p_{2,z}^{t},\ldots ,p_{20,x}^{t},p_{20,y}^{t},p_{20,z}^{t}\right]}^{T},$$

where ($p_{i,\;x}^{t}$, $p_{i,\;y}^{t}$, $p_{i,\;z}^{t}$) denotes the normalized skeletal position of the *i*th joint. The first joint, i.e., the hip center joint, is removed because, after normalization, it has been the origin of the new coordinate system and its position is always equal to (0, 0, 0).

We propose a motion energy function (MEF) to describe the motion characteristic of a skeletal sub-sequence ${\left\{st\right\}}_{a}^{b}$ (*t* = *a*, *a* + 1, …, *b*) with:

(16) $$\mathrm{MEF}({\left\{s_{t}\right\}}_{a}^{b})=\frac{1}{b-a}\sum_{j=a}^{b-1}||s_{j+1}-s_{j}|{|}_{2}^{2}.$$

The MEF quantitatively measures the motion intensity of ${\left\{st\right\}}_{a}^{b}$. We can infer that the action primitive corresponding to ${\left\{st\right\}}_{a}^{b}$ is dynamic or static through MEF(${\left\{st\right\}}_{a}^{b}$). For a static action, it changes slowly and to perform action recognition, more frames are needed so that the evidence becomes clear enough. Conversely, for a dynamic action, the action label should be output earlier due to the rapid movement. We introduce a constraint of the maximum sub-sequence length *l_max_* to ensure an acceptable delay for static actions, and a constraint of the minimum sub-sequence length *l_min_* to ensure adequate frames for generating a depth motion map. Similar length constraints can be found in existing work on human motion segmentation [27,28] and online action recognition [12,24], but our MEF-based segmentation method is very different from them. Algorithm 1 gives a full description of the offline segmentation process at the training stage.

**Algorithm 1** Offline segmentation
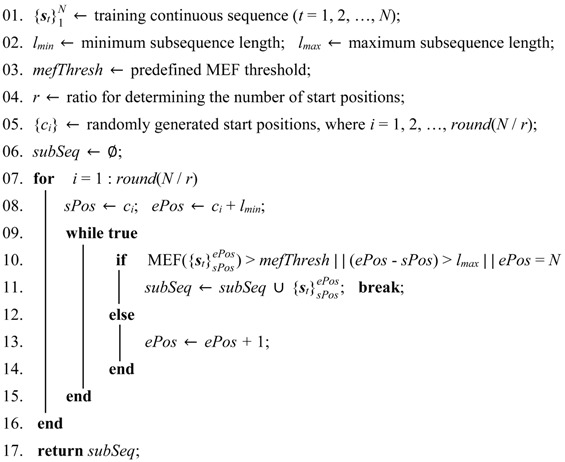


The basic idea of Algorithm 1 is that each training sequence generates several sub-sequences to represent as various action primitives as possible. At the test stage, a stream sequence should be sequentially segmented as the motion proceeds, instead of the random segmentation as described in Algorithm 1. The details of our online segmentation process are given in Algorithm 2.

**Algorithm 2** Online segmentation
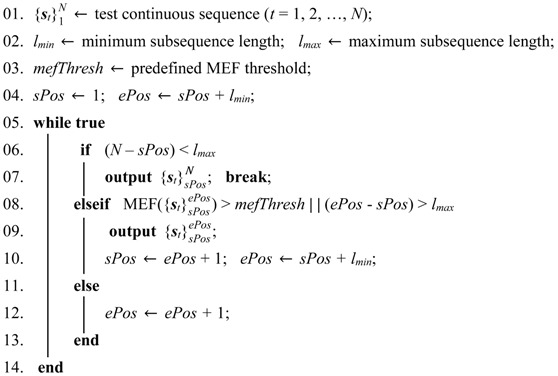


In Algorithms 1 and 2, *l_min_*, *l_max_*, *r*, and *mefThresh* are experimentally set to be 20, 50, 10, and 0.1, respectively.

#### 3.3.4. Feature Fusion

Given a segmented sub-sequence, we need to encode it as a single-feature vector. First, the sub-sequence generates three depth motion maps corresponding to front, side, and top projection views, as described in Section 3.3.1. Bicubic interpolation is then used to resize the depth motion maps to a fixed size under the same projection view for the purpose of reducing the intra-class variation and making classification. In this way, the three depth motion maps are associated with a feature vector **h** by concatenating the vectorized depth motion maps, i.e.:

(17) $$h={\left[\mathrm{vec}(\bar{{\mathrm{DMM}}_{f}}),\;\mathrm{vec}(\bar{{\mathrm{DMM}}_{s}}),\;\mathrm{vec}(\bar{{\mathrm{DMM}}_{t}})\right]}^{T},$$

where vec(·) indicates the vectorization operator.

In addition to the raw depth motion map, we also attempt the LBP-encoded depth motion map, which is inspired by [18]. In this case, a sub-sequence is encoded as:

(18) $$q={\left[lbp_{f}^{T},\;lbp_{s}^{T},\;lbp_{t}^{T}\right]}^{T},$$

where **lbp***_v_* is the LBP vector extracted from the DMM*_v_*. The LBP [40] is a simple yet effective gray scale and rotation invariant local texture operator that has been widely used. It detects “uniform” patterns in circular neighborhoods of any quantization of the angular space and at any spatial resolution with decimal numbers. The edges in the LBP-coded image are more enhanced compared with the original one, resulting in a more compact feature. The reason that we take the LBP into consideration is conditioned on its high computational efficiency [40]. We omit the LBP encoding process; readers can refer to [18] for details.

The skeletal position and velocity are incorporated as the complementary features, where the skeletal position **s***_t_* is defined as Equation (15), and the skeletal velocity is defined as the difference of two neighbor skeletal positions, i.e., **v***_t_* = **s***_t_*_+1_ − **s***_t_*. We adjust ${\left\{st\right\}}_{a}^{b}$ and ${\left\{vt\right\}}_{a}^{b}$ to a fixed length by linear interpolation along each coordinate, so that different sub-sequences have the same dimension. The reference value of the fixed length is *l_max_*. The concatenated skeletal position and velocity feature vector is:

(19) $$g={\left[s_{1}^{T},\;s_{2}^{T},\ldots ,\;s_{l}^{T},\;v_{1}^{T},\;v_{2}^{T},\ldots ,\;v_{l}^{T}\right]}^{T}.$$

To gain computational efficiency, the PCA is applied to reduce the feature dimensionality. The PCA transform matrix is calculated using the training features and then applied to the test features. Considering the incompatibility of different features, the PCA transforms of **h** (or **q**) and **g** are separately computed. Finally, each sub-sequence is encoded as a single vector by concatenating the PCA transformed **h** (or **q**) and **g**. The feature fusion and dimensionality reduction process is intuitively summarized in Figure 3.

We performed experiments on the MSR Daily Activity 3D dataset, and found that the skeletal position and velocity improve the frame-level accuracy with 12%, on average. This demonstrates the effectiveness of our solutions to extend the applications of the depth motion map.

#### 3.3.5. Feature Classification

We attempt a linear SVM and a Random Forest (RF) to accomplish the task of classification, because both of the two classifiers work fast and can satisfy the general real-time requirement. There are four derived methods in total, where the two methods without LBP encoding are denoted as DMM & SVM and DMM & RF, and the two methods with LBP encoding as LBP & SVM and LBP & RF. In our experiments, the LBP and depth motion map are implemented using the source code provided in [17,18], and the SVM using the public library LIBSVM [41].

## 4. Experiments

There have been many public 3D action recognition datasets for comparative evaluation. Zhang et al. [42] recently provided a comprehensive review on the most commonly used benchmark datasets, including 27 single-view datasets, 10 multi-view datasets, and seven multi-person datasets. Strictly speaking, among the 27 single-view datasets, the MSR 3D Online Action dataset is the only one published for supervised online action recognition. This further validates our previous statement that the amount of current research on online action recognition is relatively small.

Yu et al. [24] and Zhu et al. [12] utilized the MSR Daily Activity 3D dataset to evaluate their online action recognition performance. In fact, this dataset is originally published for offline action recognition, so no stream sequence that contains multiple actions is available. To make a comparison with [12,24], we also evaluate our methods on this dataset. Following [12,24], we concatenate all the test sequences to simulate a stream sequence.

### 4.1. MSR 3D Online Action Dataset

Detailed information on the MSR 3D Online Action dataset is listed in Table 1.

Yu et al. introduced the MSR 3D Online Action dataset in [24], and suggested to perform three experiments. The first experiment is same-environment action recognition with two-fold validation: (a) S1 for training and S2 for test, and (b) S2 for training and S1 for test. The second experiment is a cross-environment action recognition with S1+S2 for training and S3 for testing. The third experiment is continuous action recognition with S0+S1+S2 for training and S4 for testing. The first and second experiments are designed for offline action recognition, so all the sequences in the two experiments have been segmented and contain only one action. The third experiment is designed for online action recognition, so each test stream sequence contains multiple actions.

#### 4.1.1. Quantitative Comparison

Our methods are developed for online action recognition and operate on a stream sequence. Hence, only the third experiment is suitable for evaluating our methods. Following Yu et al. [24], the frame-level accuracy is used as the online performance measure, i.e., the percentage of frames that are correctly classified. Note that the frame-level accuracy is different from the accuracy of offline action recognition that is defined as the ratio of correctly classified segmented sequences to total segmented sequences. Table 2 lists the frame-level accuracies of our methods and the baseline methods. The best result is in bold. In the whole paper, the DSTIP + DCSF, EigenJoints, and Moving Pose are implemented by Yu et al. and, accordingly, all the accuracies of the three baseline methods are reported by them in [24].

We can see that the DNBC works best among all the methods. For the Orderlet [24], we remind readers a fact that it relies on manually labeled data to describe action-related object positions: at the training stage, the object in each frame is manually labeled with a bounding box, and the distance between the hand and the object center is used to model the frequent hand-object shifts. The other baseline methods and our methods are weakly supervised in that all the features are automatically extracted. The manually labeled data are indeed helpful to model action discriminations. However, they may not be friendly, or even acceptable, in practical scenes, since processing them is a very laborious and time-consuming job for users.

Among the first type of methods, the K-SVD & Lasso performs worst, but compared to the weakly supervised baseline methods, it still works better than the EigenJoints and DSTIP + DCSF. Both the K-SVD & Ridge and K-SVD & Elastic Net outperform the DSTIP + DCSF, EigenJoints, and Moving Pose, but achieve lower accuracies than the Orderlet and DNBC.

The reasons that the Ridge and Elastic Net outperform the Lasso can be explained from the aspect of the predictor selection principle. For the Ridge, its collaborative representation mechanism maybe helps it to work better than the Lasso [43]. Zou et al. [33] point out that, if highly correlated predictors exist, the Lasso is inclined to select a few of them. In the extreme situation where some predictors are exactly identical, the Lasso does not ensure assigning identical coefficients to them. However, the fact is that highly-correlated predictors universally exist in the same dictionary, because they are extracted from the training sequences of the same action. In contrast, the Elastic Net possess a grouping effect to simultaneously select the correlated predictors [33], thereby resulting in a better reconstruction error and confidence.

For the second type of methods, we conclude that the LBP encoding works better than the direct use of the depth motion map, and the SVM works better than the RF. Among our methods, the best result is achieved by the LBP & SVM. Although the Orderlet additionally introduces the manually-labeled data, both the LBP & SVM and DMM & SVM outperform it. We can also see that our second method achieves much higher accuracy than the EigenJoints, DSTIP + DCSF, and Moving Pose.

It is not difficult to see that our second method remarkably outperforms the first type, which demonstrates that the depth motion map combined with the skeletal position and velocity is more effective than the pairwise relative joint position combined with the LOP. Perhaps the causes are two-fold: (i) the depth motion map characterizes an action primitive by extracting features from a sub-sequence, which is more informative than the frame-wise position and LOP descriptor, although the temporal smoothness scheme is introduced. (ii) The temporal order is not sufficiently considered in the first method: both the learned dictionaries and the sliding windows do not take the frame order into account.

Our best method LBP & SVM achieves a lower accuracy than the DNBC [36] with 2.7%. The causes are three-fold: (i) the DNBC is built under a time series framework, which is more descriptive in characterizing temporal motion trajectories. (ii) Compared to the LBP encoded depth motion map, the pose-based shape adopted by the DNBC is a more precise descriptor for modeling the spatial configuration of human body parts. (iii) The DNBC simultaneously considers the object manipulation, whereas the LBP & SVM mainly characterizes the human pose and movement. However, the DNBC may have the problem of real-time implementation, because it needs to perform interpolation in the Riemannian shape space. In addition, its computational complexity is strongly influenced by the number of actions. The DNBC runs with only seven FPS [36] on the MSR 3D Online Action Dataset that contains only seven actions. In contrast, the LBP & SVM performs action recognition based on one-shot classification, which is less influenced by the number of actions. The LBP & SVM runs with 62 FPS on the same dataset. Note that the real-time performance is as important as the accuracy for online action recognition, since it also strong influences the application feasibility.

#### 4.1.2. Action Recognition Visualization

**Figure 6:**
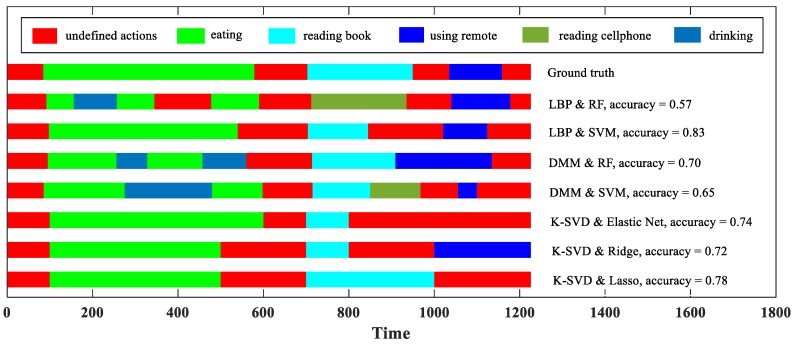
Visualization of online action recognition results.

Figure 6 visualizes an action recognition result of our methods on a stream sequence, enabling readers to intuitively understand the frame-level accuracy. In this example, the LBP & SVM correctly recognize all the seven ground truth actions, but its accuracy is only 0.83. This is because, in addition to correct action classification, online action recognition also puts forward the requirement of action detection.

From Figure 6, we can also see the respective deficiencies of our methods. Due to the sliding windows, the first type may skip an action with a short duration, i.e., the action may be mistakenly split and incorporated into its adjacent actions. In Figure 6, the “using remote” is skipped by the K-SVD & Elastic Net. The second type may have the sudden change problem when encountering an action with a long duration. In Figure 6, “eating” is mixed with “drinking” when the DMM & RF performs action recognition. Designing an adaptive action detecting algorithm for the improvement is not an easy task, so we leave this matter for our future work.

#### 4.1.3. Temporal Smoothness

**Figure 7:**
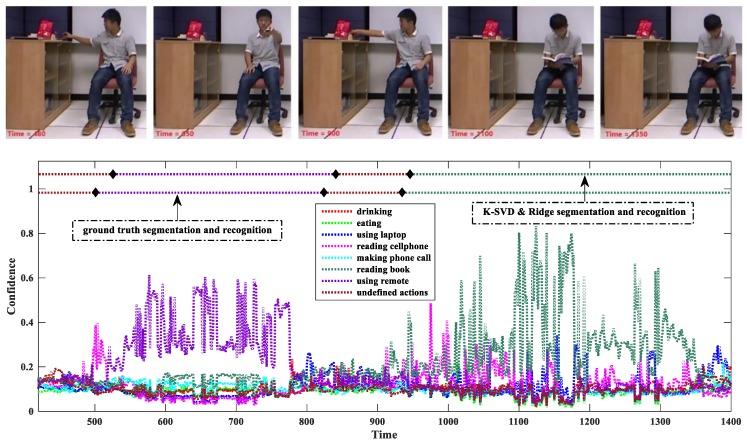
Confidence variation and temporal smoothness. Each frame is predicted by eight confidences associated with the seven meaningful actions and undefined actions. The black diamonds indicate the cut points between consecutive actions. The colors of the dashed lines across the diamonds indicate the action classes. The ground truth cut points are 500, 823, and 934. The detection cut points of the K-SVD & Ridge are 525, 840, and 945. The upper five images show the five key poses with the temporal instances 480, 650, 900, 1100, and 1350. The RGB channel and depth channel of the Kinect are not strictly synchronized, but this slight misalignment can be neglected for observation.

We experimentally studied the influence of the temporal smoothness scheme for the first method and found that on average it improved by about 11% compared to the single-frame decision Equation (13). Figure 7 shows a confidence variation as a stream sequence goes on.

In this example, the confidence is calculated by the K-SVD & Ridge. Due to the space limitation, we only plot the sub-sequence with the frame indices from 420 to 1400. The sub-sequence contains two meaningful actions, namely “using remote” and “reading books”, and two undefined actions. For visual analysis, we sample several key poses to observe, and five of them are shown in the upper images of Figure 7.

We can see that the confidence indeed measures the action membership of the test frames. When the evidence is ambiguous, the confidences of all the actions of interest are quite low, as the frames within the temporal intervals (420, 525) and (840, 945), shown in Figure 7. The continuity of the confidence facilitates rejecting recognition by simply setting a threshold, i.e., these frames are classified as undefined actions. When the evidence is clear, the confidence value of the true action is significantly larger that of any other action, as the frames within the temporal intervals (525, 840) and (945, 1400) shown in Figure 7.

Although the confidence of the true action is dominant on the whole, it varies sharply and its high values are not kept all the time. This validates the necessity of the temporal smoothness, because the sliding window ensures that all the frames within it have the same action label, and the average confidence makes the action recognition result more reliable.

### 4.2. MSR Daily Activity 3D Dataset

The MSR Daily Activity 3D dataset contains 16 activities: “drink”, “eat”, “read book”, “call cellphone”, “write on a paper”, “use laptop”, “use vacuum cleaner”, “cheer up”, “sit still”, “toss paper”, “play game”, “lay down on sofa”, “walk”, “play guitar”, “stand up”, and “sit down”. Each activity is performed by 10 subjects, twice each. We follow the standard cross-subject evaluation protocol: half of the subjects are used as training data, and the other half are used as test data.

#### 4.2.1. Quantitative Comparison

Table 3 lists the frame-level accuracy of different methods. We can see that the first type of our methods achieves low accuracies compared to the baseline methods, and only works better than the DSTIP + DCSF. The main cause is that the temporal smoothness scheme is not applicable on the MSR Daily Activity 3D dataset. The stream sequence in this dataset is simulated by concatenating segmented sequences, and no meaningless actions are defined. The evidence of the action class is quite ambiguous at the beginning and the end of the segmented sequences, but we cannot assign the label of undefined actions to these frames. After all, the simulated stream sequence is still different from the real stream sequence of the MSR 3D Online Action dataset. Therefore, we utilize the average confidence within a fixed-size window to predict action labels instead. To ensure our methods have the same delay as the baseline methods [10,11,20,24], the size of the window is set to be 100.

The second of our methods works much better than the first. The superiority of the SVM and the LBP remains in this experiment, although the action number and the action class of the MSR Daily Activity 3D dataset are very different from those of the MSR 3D Online Action dataset. Among all the methods, the best result is achieved by the LBP & SVM. The MEMM is most recently proposed among the baseline methods, but the second type of method performs significantly better than it, e.g., the LBP & SVM outperforms it with 7.5%.

Except the MEMM, the accuracies of the other three weakly supervised baseline methods are still not attractive. The DSTIP + DCSF and EigenJoints are originally proposed for offline action recognition, and they are implemented to perform online action recognition by Yu et al. [24]. Although the authors of the Moving Pose claimed that the Moving Pose enabled real-time action recognition, they actually only provided an observational latency analysis on segmented sequences [11]. This means that its capability of recognizing stream sequences is still unknown until Yu et al. [24] evaluate its frame-level accuracy.

The examples of the DSTIP + DCSF and EigenJoints indicate such a fact: extending offline methods to online applications is not straightforward and, at least, this does not ensure a satisfactory result. To develop an appealing online action recognition method based on existing work, it is necessary to carefully explore from various aspects, e.g., feature extraction and classifier selection. The motivation of our work just stems from this consideration.

#### 4.2.2. Confusion Matrix

**Figure 8:**
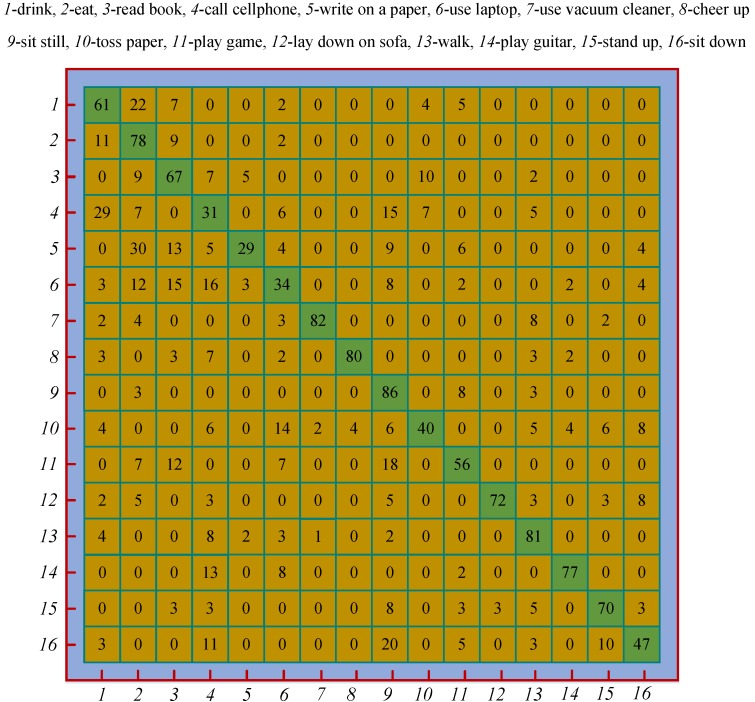
Confusion matrix of the LBP & SVM. Each row corresponds to the ground truth labels and each column corresponds to the recognition labels.

On the two datasets, the LBP & SVM achieved the best accuracy among our methods, so we took it as an example to analyze its confusion matrix, as shown in Figure 8. All the accuracies in the confusion matrix have been rounded to the nearest percent. We developed seven methods in total. Analyzing all the methods and listing all the confusion matrices will occupy too much space.

On the whole, the confusion matrix is relatively sparse, which indicates the holistic discriminative capability of the LBP & SVM. For those easily distinguishable actions, e.g., “use vacuum cleaner” and “cheer up”, the LBP & SVM achieves very high accuracies. The similar pairwise actions, e.g., “drink” vs. “call cellphone”, are easily confused each other, so the accuracies of those actions are quite low. The action with the highest accuracy is “sit still”, which forcefully demonstrates that the LBP encoded depth motion map combined with the skeletal velocity and position can effectively characterize the static actions. The difference of the actions “stand up” and “sit down” lies in the temporal order of the human poses, and the average accuracy of the two actions is 0.585. We experimentally found that the average accuracy would drop by 19% if the skeletal velocity and position feature vectors were removed from the fused feature vector. This validates the effectiveness of the two complementary features.

### 4.3. Real-Time Performance

We evaluate the real-time performance of our methods through the computational latency and the observational latency. The former means the system computational time consumed on feature extraction and classification at the test stage, which can be equivalently converted to the number of frames that the system can process per second, i.e., FPS. The latter represents the number of frames that have been observed before performing action recognition.

For the computational latency, the FPS of our methods and the baseline methods are listed in Table 4. The influence of the computer configuration is neglected. Our methods are implemented on a normal desktop with the mixed programming between MATLAB and C languages. We can see that our methods satisfy the basic real-time requirement.

The observational latency of the first method is determined by the size of the two windows in the temporal smoothness scheme. In our experiments, the maximum size of the two windows is set to be 50, so the maximum delay is 100 frames. All the reported accuracies of the DSTIP + DCSF, EigenJoints, and Moving Pose are also conditioned on a 100-frame delay [24]. For the second type, the observational latency is determined by the constraints *l_min_* and *l_max_*. Since *l_max_* is set to be 50, the maximum 50-frame delay is less than the baseline methods.

## 5. Conclusions

We developed two methods of online action recognition. The first utilizes the K-SVD to extract class-specific dictionaries based on the pairwise relative joint positions and LOP features. In conjunction with the learned dictionaries, frame-wise action recognition is achieved by the Lasso, Ridge, and Elastic Net. A temporal smoothness scheme is proposed to prevent the jitter of the predicted action labels.

The second type starts from the depth motion map. In view of the fact that the depth motion map works on a segmented sequence, we propose an offline segmentation algorithm for training, and an online segmentation algorithm for testing. Since the depth motion map has a limited discriminative power for static actions, we introduce the skeletal position. The depth motion map loses the temporal information, resulting in it being incapable of distinguishing actions whose differences only lie in the temporal order of human poses. For this problem, the skeletal velocity is incorporated to the feature vectors. The two sequence segmentation algorithms and the two complementary descriptors are experimentally proved to be effective.

Our methods achieve state-of-the-art accuracy, which demonstrates the significance of our work. Since the second type of method works better than the first, the second type will become the research emphasis of our future work. We intend to improve our sequence segmentation algorithms by exploring an adaptive detection approach that is able to find action primitives with middle-level or high-level semantics. The importance of the adaptive detection approach is that it cannot only efficiently mine discriminative action primitives for training, but also prevents the jitter when predicting actions with a long duration.

## Figures and Tables

**Table 1:** MSR 3D Online Action dataset.

Action Subset	Description (The Number *#i* is the Action Category Index)
S0	This subset contains background actions (#10, or undefined actions), which are only used for continuous action recognition. There are 14 sequences in total.
S1	This subset contains seven meaningful actions, including drinking (#0), eating (#1), using laptop (#2), reading cellphone (#3), making phone call (#4), reading book (#5), and using remote (#6). There are 112 sequences in total, and each sequence contains only one action.
S2	The actions in this subset are the same as in S1 except that the human subjects change. There are 112 sequences in total, and each sequence contains only one action.
S3	The actions in this subset are the same as in S1 and S2, but both the action execution environment and human subjects change. There are 112 sequences in total, and each sequence contains only one action.
S4	There are 36 unsegmented action sequences (#8), and each one contains multiple actions. The meaningful actions (#0-#6), as well as the background actions (#10), are recorded continuously. The duration of these sequences lasts from 30 s to 2 min. For evaluation purpose, each frame is manually labeled, but the boundary between two consecutive actions may not be very accurate since it is difficult to determine the boundary.

**Table 2:** Comparison on the MSR 3D Online Action dataset.

Method		Accuracy
Ours	K-SVD & Lasso	0.433
	K-SVD & Ridge	0.506
	K-SVD & Elastic Net	0.528
	DMM & SVM	0.571
	DMM & RF	0.532
	LBP & SVM	0.582
	LBP & RF	0.543
	Orderlet [24]	0.564
	DSTIP + DCSF [20]	0.321
	Moving Pose [11]	0.236
	EigenJoints [10]	0.500
	DNBC [36]	0.609

**Table 3:** Comparison on the MSR Daily Activity 3D dataset.

Method		Accuracy
Ours	K-SVD & Lasso	0.375
	K-SVD & Ridge	0.413
	K-SVD & Elastic Net	0.426
	DMM & SVM	0.613
	DMM & RF	0.564
	LBP & SVM	0.622
	LBP & RF	0.596
	MEMM [12]	0.547
	Orderlet [24]	0.601
	DSTIP + DCSF [20]	0.246
	EigenJoints [10]	0.470
	Moving Pose [11]	0.452

**Table 4:** Comparison of the computational latency.

Method		FPS
Ours	K-SVD & Lasso	482
	K-SVD & Ridge	34
	K-SVD & Elastic Net	27
	DMM & SVM	87
	DMM & RF	215
	LBP & SVM	62
	LBP & RF	159
	MEMM [12]	10
	Orderlet [24]	25
	DNBC [36]	7
